# Radix saw: a useful tool for rhinoplasty to correct high radix

**DOI:** 10.1016/j.bjorl.2019.06.013

**Published:** 2019-08-07

**Authors:** Süreyya Şeneldir, Denizhan Dizdar, Altuğ Tuna

**Affiliations:** a Süreyya Şeneldir Clinic, Istanbul, Turkey; b İstinye University, Faculty of Medicine, Bahçelievler Medical Park Hospital, Department of Otolaryngology, İstanbul, Turkey; c Private Office, Frankfurt, Germany

**Keywords:** Rhinoplasty, Radix, Nose

## Abstract

**Introduction:**

The most difficult aspect of radix lowering is determining the maximum amount of bone that can be removed with osteotomes; here, we describe use of a radix saw, which is a new tool for determining this amount.

**Objective:**

In this study, we describe use of a radix saw, which is a new tool to reduce the radix.

**Methods:**

The medical charts of 96 patients undergoing surgery to lower a high radix between 2016 and 2017 were assessed retrospectively. All operations were performed by the senior surgeon. Outcomes were assessed by comparing preoperative photographs with the most recent follow-up photographs (minimum of 6 months postoperatively). The photographs were all taken using the same imaging settings, and with consistent subject distance and angulation. The photographs were subsequently analysed by authors.

**Results:**

The study population consisted of 96 patients (70 women, 26 men) who underwent rhinoplasty between 2016 and 2017. The mean age of the patients was 28.8 years (range: 18–50 years) and the mean clinical follow-up period was 1.8 years. No patient required revision surgery due to radix problems, and there were no cases with unwanted bone fragments or radix asymmetry. The swelling and oedema seen immediately after surgery subsided after an average of 7–10 days.

**Conclusion:**

In conclusion, a radix saw can be used for rhinoplasty requiring delicate bone removal in patients with a high radix. Level IV: Evidence obtained from multiple time series with or without the intervention, such as case studies. Dramatic results in uncontrolled trials might also be regarded as this type of evidence.

## Introduction

The radix is a depression at the root of the nose, which defines the nasal root and the origin of the nose from the point of the glabella. The radix extends inferiorly from the nasion to the level of a horizontal line passing through the lateral canthi, and superiorly from the nasion for an equivalent distance.^1^

Radix localisation can be performed using several methods. The radix defines the most concave part of the cephalic dorsum. The normal distance from the radix to the inner canthus is 6 mm and the distance between the corneal plane and the radix plane is about 9–14 mm.^1^, ^2^, ^3^, ^4^ Radix position is evaluated in the cephalocaudal and anteroposterior axes. The normal position of the radix in the cephalocaudal axis is between the level of the upper lid margin and the supratarsal fold with the eyes looking straight ahead. Lower and higher radix positions in both axes not only affect the aesthetic appearance of the nose, but also the nasal base and apparent length.^4^

The position of the radix significantly influences the overall balance of the nasal profile; it impacts the nasal contour, length, angulation and height.

A low radix position results in reduced nasal projection, increased tip projection, and a nasal base that appears larger than its actual size. A high radix may be seen in some patients who seek rhinoplasty. In patients with a high radix, the radix projection is above the normal limit, such that the nose appears excessively long. In the profile view, the nose-to-forehead angle may be wide, such that the nose and forehead appear continuous, an effect known as “avatar nose”. Alternatively, the nasal bones may be situated apart from each other, leading to the appearance of excessively wide spacing of the eyes.

The most difficult aspect of radix lowering is determining the maximum amount of bone that can be removed with osteotomes; piezoelectric and classical osteotomies are widely used.^5^, ^6^, ^7^ Here we describe use of a radix saw, which is a new tool for determining this amount.

## Methods

This retrospective case series study was performed in accordance with the Declaration of Helsinki. Informed consent was provided by all patients following institutional ethics board approval (Istanbul Education and Research Hospital Ethics Board no: 1538). The medical charts of 96 patients undergoing surgery to lower a high radix between 2016 and 2017 were assessed retrospectively. All operations were performed by the senior surgeon.

**Figure 1:**
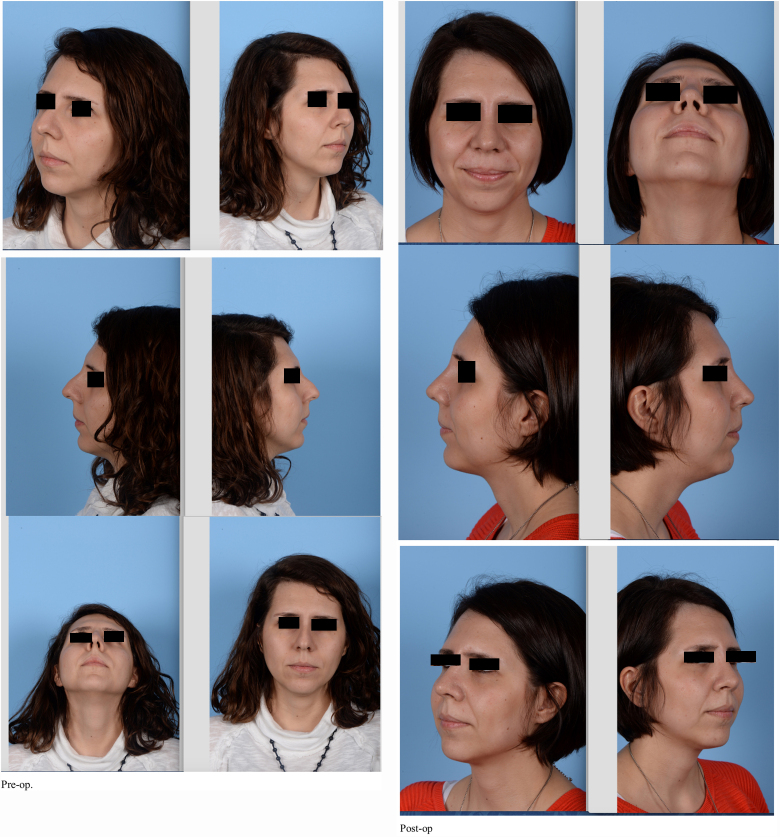
Patient 1. Pre and post-op photos.

**Figure 2:**
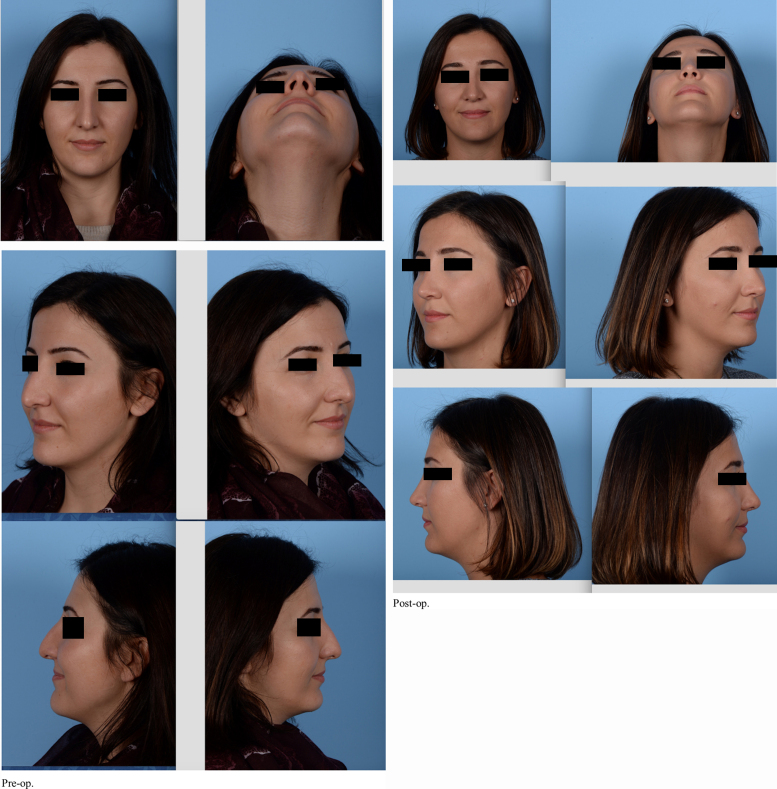
Patient 2. Pre and post-op photos.

**Figure 3:**
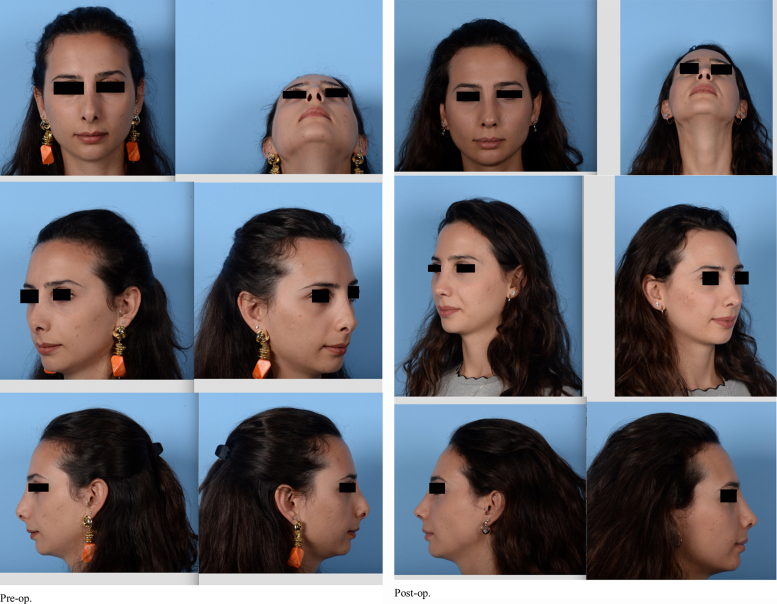
Patient 3. Pre and post-op photos.

Outcomes were assessed by comparing preoperative photographs with the most recent follow-up photographs (minimum of 6 months postoperatively). The photographs were all taken using the same imaging settings, and with consistent subject distance and angulation; from 1.5 m; frontal, 45 degree, profile, basal angles. A Nikon d800 camera was used, with 100 mm, f14, macro lens, İSO 100, double paraflash from 1.5 m. The photographs were subsequently analysed by the senior author (Figure 1, Figure 2, Figure 3). The outcomes were also assessed by asking the patients’ opinions on their own radix position and shape. Radix asymmetr yand unwanted bone fragments were evaluated. The postoperative oedema was assessed with physical examination.

Patients were selected from a database of rhinoplasty cases containing information regarding patient demographics, preoperative analyses, operative techniques, and postoperative outcomes and complications.

### Radix saw

**Figure 4:**
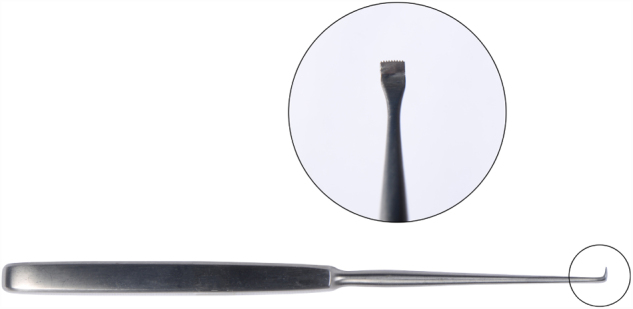
Radix saw.

The radix saw was made of hardened stainless steel and has a tip width of 4 mm, depth of 7 mm, gear angle of 90° and shank length of 12 cm. The head of the instrument was small, such that it did not require excessive soft tissue elevation and did not cause bruising or reddening of the skin (Fig. 4). The tool was created by the senior author.

### Surgical technique

In a sitting posture on the operating table (to avoid any influence of gravity), the actual radix position and depth of the desired position was measured and marked on the skin. After general with infiltration anaesthesia, the dorsum was reached by supraperichondrial dissection (via subcutaneous dissection) with an inverse V-shaped columellar incision if open technique was used or the dorsum could also be reached via intercartilagineous incision. For both open and closed technique the operative steps were as follows. The skin and soft tissue envelope were dissected and raised in the supraperichondrial plane to the rhinion. Subsequent subperiosteal elevation was performed over the nasal bones.

**Figure 5:**
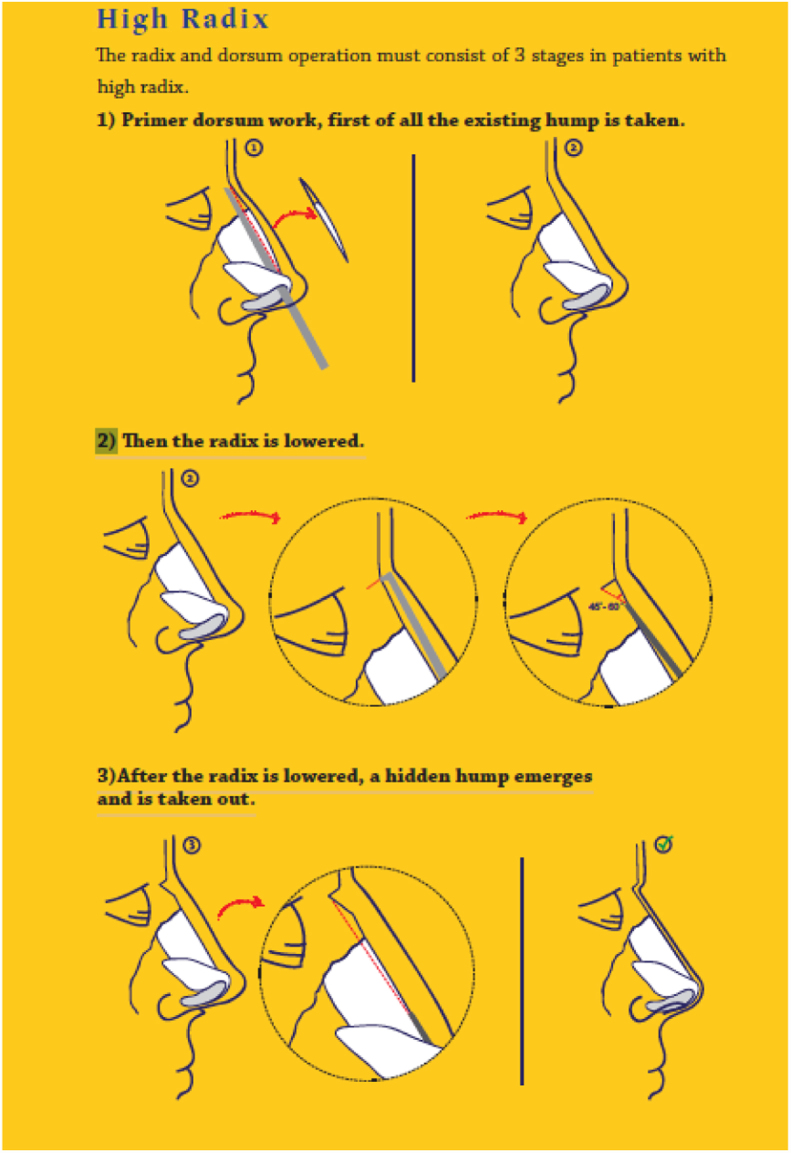
Steps of procedure.

The radix and dorsum operation consisted of three stages in patients with a high radix: (1) initial work on any existing hump in the nasal dorsum; (2) lowering of the radix; (3) removal of any residual hump following radix lowering (Fig. 5).

It was important to pay attention to the order of steps. First the existing primary hump was lowered using standard ostetomes or rasps. Than the ideal radix position was determined by making a straight and non-traumatic incision in the nasal bone, which required fine wrist movement, using a radix saw in the vertical plane. The depth of the radix was adjusted by varying the bone incision depth with the radix saw, up to a maximum depth of 7 mm (i.e. the length of the saw). No pressure should have been applied to the saw at this point. However, the borders of the medial canthus could be palpated and supported laterally.

**Figure 6:**
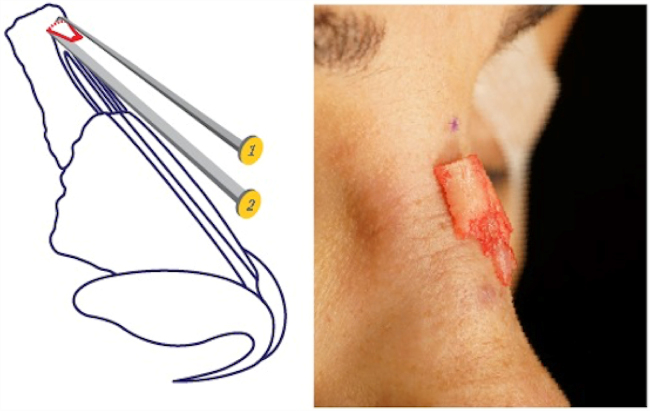
(1) Radix saw; (2) straight osteotome.

**Figure 7:**
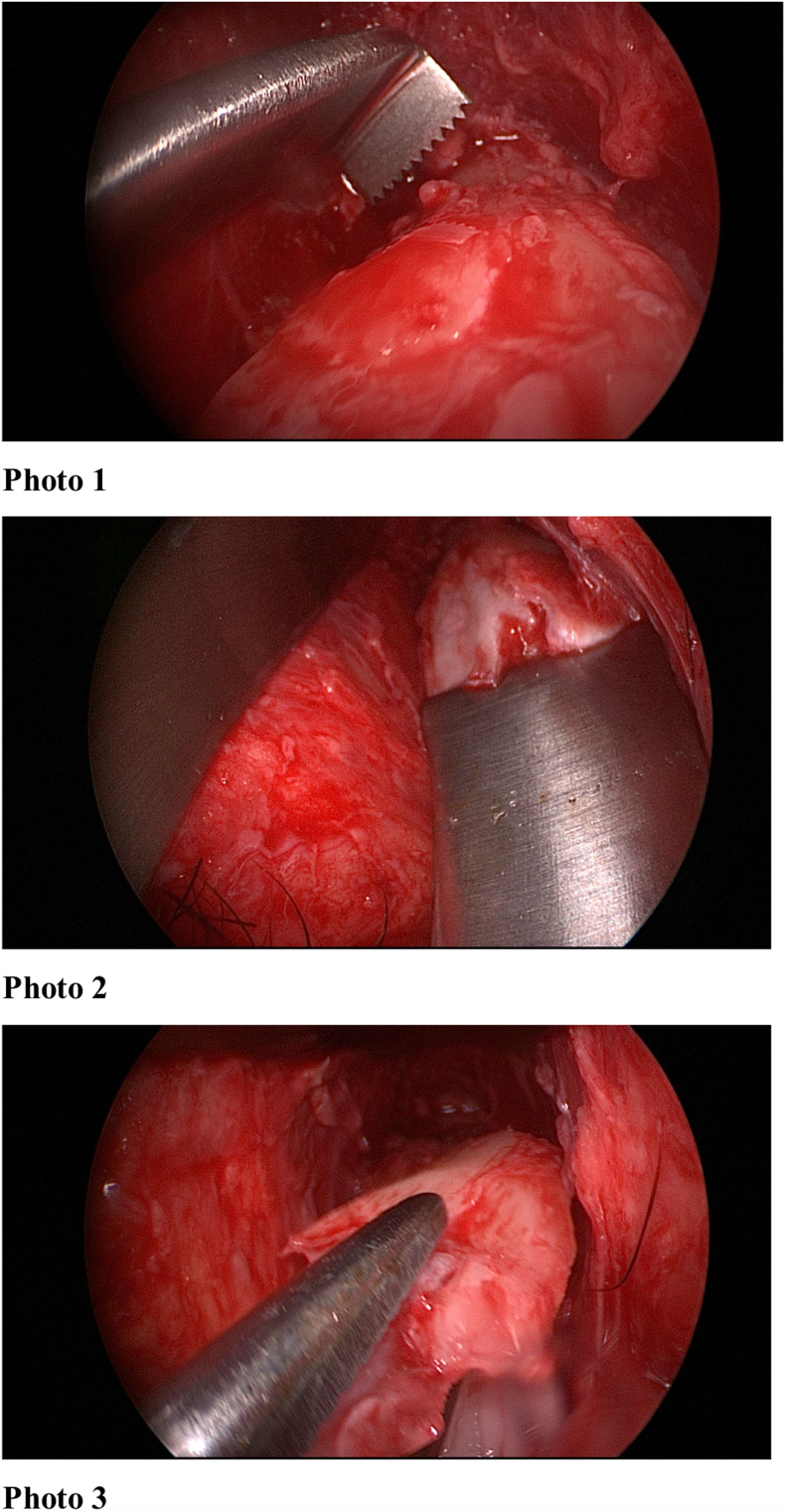
Per-op endoscopic view.

Thus, the upper limit of the bone to be removed was determined and cut. Then, a straight osteotome was placed in the radix area at a 40/60 (Fig. 6) degree angle and a bone incision was made that reaches the transverse osteotomy above. After the incisions were finished, the wedge-shaped bone was released. Controlled incisions and resection were performed with the use of an osteotome and a saw. If necessary more bone could be removed with a rasp. At this point the radix saw could also be used as a rasp. Care was required to avoid over-resection. Reduction should have been performed using the saw as a rasp tool, to ensure minimal resection, irregularity and dorsal reduction. In this manner, the radix saw could be used as an alternative to a file, to avoid scraping of the radix in the caudal direction and ensure radix alignment according to the pre-determined dorsum height (Supplementary Material 1) (Fig. 7).

After the radix and dorsum were modified as desired, the operations is continued with standart lateral osteotomies and tipplasty.

## Results

The study population consisted of 96 patients (70 women, 26 men) who underwent rhinoplasty between 2016 and 2017. Ten patients were operated with closed technique; other 86 were operated with open technique. The mean age of the patients was 28.8 years (range: 18–50 years) and the mean clinical follow-up period was 1.8 years. The mean long-term follow-up period was 1.6 years. Direct follow-up was available for 96 patients after 1 year; they were asked if they were satisfied with the radix points and they were all had physical examination of the radix area.

Over the 2 year study period 95 patients were satisfied with the radix points and physical examination showed no problem in the radix area; 1 patient (<1%) presented to the author with complaints about the bony dorsum near the radix. The cause of dissatisfaction in this patient were minimal bony prominences.

Two patients had revision surgeries due to tip problems. These revisions were due to tip graft visibility and tip asymmetry. The oedema and swelling was evaluated with physical examination. The swelling and oedema seen immediately after surgery subsided after an average of 7–10 days. No undesirable oedema was observed after 10 days postoperatively.

## Discussion

Two parameters must be known to identify the ideal radix position:

1) The ideal vertical position of the radix: this is the point at which a horizontal line drawn from the middle of the upper eyelid intersects the centerline of the nose. The radix position affects nose length: whereas a caudally located radix results in a short nose, in patients with a more cephalic radix position the nose will be long. The former occurs more frequently.
2) The ideal radix projection (height) in the anterior coronal is 9–14 mm. Radix projection is important as it directly affects the extent of nasal projection. The radix and dorsum heights must be in balance. To establish a good balance, the radix height must first be ideal, i.e. 9–11 mm in women. Men require a stronger dorsum, and the radix height must be at the upper limit of ideal for patients with thicker skin.^8^, ^9^

Most techniques used to reduce the radix focus on lowering the nasal bones and the results largely depend on the status of the nasal skin, subcutaneous tissues and muscles (such as the procerus and corrugator supercilii). A combination of thick skin and tissue softness makes it difficult to precisely determine the extent of radix reduction or augmentation required.

All measurements of the radix should be made while the patient is in a sitting position, and planning should be performed with this in mind. When measurements are made with the patient lying down, gravity can negatively affect the radix position.

Various techniques have been proposed for removal of bone from the radix region, including rasping, osteotomy and use of a burr.^10^, ^11^, ^12^, ^13^ Osteotomy is the preferred method for dealing with a high radix accompanied by a moderate to severe bony hump. Its advantages include en bloc resection of the bone without residual bony particles, while drawbacks include a risk of over-resection and asymmetrical bone removal. It is also possible to achieve a clear break with osteotomy. Rasping can facilitate step-by-step removal of excess bone, but it causes severe trauma to the soft tissue and is not suitable for cases with a very high radix. Another disadvantage of rasping is its imprecision regarding radix position. Various osteotomes have been used to deepen the nasofrontal angle. Flat osteotomes used together with a radix saw have been suggested to weaken the bony root. Quisling and Rubin osteotomes do not have the same high degree of accuracy associated with percutaneous osteotomy.

Guyuron was the first to use a guarded burr for deepening of the radix, which makes conservative bone removal possible. The complications associated with use of a burr include a risks of seroma and penetration of the frontal sinus.^12^

Piezoelectric systems which are used commonly can be used to reduce radix but, because the area must be washed with saline, that may affect the perception of the radix and oedema can occur after the use of piezo.^13^

The soft tissue of the radix occasionally requires attention. In such cases, it is recommended to perform the dissection superficial to the muscle and remove the bone with the procerus muscle still attached. It is necessary to exert constant pressure on the area for a few days to avoid haematoma, fibrosis and scar formation. The use of a specially designed splint may be considered.

A number of rhinoplasty surgeons have developed techniques to lower the radix, but no procedures capable of thinning the skin envelope over the radix have yet been reported. Although minor reductions in the caudal portion of the radix may be achieved using a rasp, substantial reduction requires a more aggressive approach. The use of an osteotome to perform wedge resection of the nasofrontal bones may effectively reduce the projection of the radix and deepen the nasofrontal angle. If the radix is overdeveloped and thus makes a significant contribution to the nasofrontal angle, a guarded burr can be used to reduce the bone. Guyuron suggested that the bone height must be reduced by at least 4–5 mm to afford a visible reduction of 1 mm. However, this is not possible in all patients due to variations in frontal bone anatomy. Removal of nasofrontal bone at the level of the radix leads to a visible reduction in bone height of only approximately 25%.^14^, ^15^, ^16^

Nasal skin thickness differs among the various nasal regions. Given that overprojection in the area of the radix may not be attributable only to bone, manipulations of only the nasal bones may not yield an ideal solution.^17^

The radix saw has a number of advantages; for example, it is possible to mark the upper limit with respect to the amount of bone to be removed and, by using osteotomes thereafter, the bone can be smoothly removed and the ideal radix position precisely determined. Use of a radix saw is associated with less oedema and swelling, as the cutting of the bone can be precisely controlled, in addition to a reduction in bony irregularities and, in cases with a small hump, direct removal thereof.

## Conclusion

A radix saw can be used for rhinoplasty requiring delicate bone work in patients with a high radix.

The English in this document has been checked by at least two professional editors, both native speakers of English. For a certificate, please see: http://www.textcheck.com/certificate/PaBdx8.

## Conflicts of interest

The authors declare no conflicts of interest.
